# Cognition, The Menstrual Cycle, and Premenstrual Disorders: A Review

**DOI:** 10.3390/brainsci10040198

**Published:** 2020-03-27

**Authors:** Jessica Le, Natalie Thomas, Caroline Gurvich

**Affiliations:** Monash Alfred Psychiatry Research Centre, Central Clinical School, Monash University and The Alfred Hospital, Melbourne, VIC 3004, Australia; jle30@student.monash.edu (J.L.); natalie.thomas@monash.edu (N.T.)

**Keywords:** cognition, menstrual cycle, premenstrual mood disorders, premenstrual syndrome (PMS), premenstrual dysphoric disorder (PMDD)

## Abstract

Sex hormones, such as estrogens, progesterone, and testosterone, have a significant influence on brain, behavior, and cognitive functioning. The menstrual cycle has been a convenient model to examine how subtle fluctuations of these hormones can relate to emotional and cognitive functioning. The aim of the current paper is to provide a narrative review of studies investigating cognitive functioning in association with the menstrual cycle in biological females, with a focus on studies that have investigated cognitive functioning across the menstrual cycle in females with premenstrual mood disorders, such as premenstrual syndrome (PMS) and premenstrual dysphoric disorder (PMDD). In line with previous reviews, the current review concluded that there is a lack of consistent findings regarding cognitive functioning across the menstrual cycle. Most studies focused on changes in levels of blood estrogen, and neglected to explore the role of other hormones, such as progesterone, on cognitive functioning. Cognitive research involving premenstrual disorders is in its infancy, and it remains unclear whether any cognitive disturbances that are identified may be attributed to negative experience of mood and psychological symptoms or be a more direct effect of hormonal dysregulation or sensitivity. Suggestions for future research are provided.

## 1. Introduction

Sex steroid hormones, such as estrogens, progesterone, and testosterone influence cognitive functioning and have been at the basis of studies examining sex differences in behavior for decades. The menstrual cycle is a model of hormonal flux that has garnered interest in understanding hormonal influences on emotional and cognitive functioning. The influence of sex hormones on cognitive functioning has primarily been driven by sex differences historically demonstrated in certain cognitive tasks. More recently, menstrual cycle phase-based assessments of cognition have been applied to clinically-relevant disorders such as premenstrual syndrome (PMS) and premenstrual dysphoric disorder (PMDD). Both of these disorders have significant psychosocial impacts in the week(s) leading up to menstruation, but the cognitive aspects of these disorders have not been fully explored. This paper is a narrative review of current literature examining cognitive functioning over the menstrual cycle, with a specific focus on determining the current understanding of cognition in PMS and PMDD.

## 2. The Menstrual Cycle and Premenstrual Disorders

The menstrual cycle is the physiological process in which sex hormone fluctuations alter the uterus lining and drive ovarian production of eggs so reproduction can occur [1]. The hormonal fluctuations of the menstrual cycle are regulated by the hypothalamus–pituitary–gonadal (HPG) axis. The hypothalamus secretes gonadotropin-releasing hormone (GnRH), which stimulates the anterior pituitary to produce luteinizing hormone (LH) and follicle stimulating hormone (FSH). Both LH and FSH stimulate the ovaries in females to produce estrogen and progesterone. Hormone levels then negatively feed back to the hypothalamus and pituitary to decrease GnRH, LH, and FSH secretion.

For women of reproductive age with regular cycles, cycle length is normally between 24–35 days [2]. There are two broad phases, the follicular phase and the luteal phase. The follicular phase starts on day 1 of menses and ends with ovulation, followed by the luteal phase until menses occurs again, when the cycle repeats. In the first week (the early follicular phase) estradiol and progesterone are typically at low levels. In the following week, the late follicular phase, estradiol rises rapidly, and just before ovulation, there is an LH surge. In the luteal phase, estradiol falls sharply then starts to rise again, while progesterone rises to peak at day 21 in a 28-day cycle. Both hormone levels then gradually fall in the late luteal phase before menses.

The psychological effects of the menstrual cycle have received increasing interest particularly due to their clinical relevance in premenstrual disorders such as PMS and PMDD. Premenstrual psychological changes can include emotional changes, such as irritability, mood lability, or depressed mood, as well as cognitive symptoms such as difficulties concentrating [3]. While PMS generally refers to any somatic or psychological symptoms that impact functioning [4], PMDD is a newly- recognised psychiatric disorder which afflicts 1–8% of women [5,6], often conceptually placed at the extreme end of premenstrual symptom severity. PMDD has strict diagnostic criteria which were implemented into the American Psychiatric Association’s Diagnostic and Statistical Manual (DSM-5) in 2013 [3]. While the etiology remains unknown, the general consensus is that premenstrual symptoms are due to an increased central nervous system sensitivity to menstrual cycle hormone fluctuations [7,8]. Symptoms generally occur cyclically in the late luteal phase of the menstrual cycle, and remit once menses occurs in the follicular phase. Possible mechanisms involved include dysregulation of progesterone’s main metabolite, allopregnanolone, in combination with serotonergic and gamma aminobutyric acid (GABA)ergic pathways [7,8].

## 3. Sex Hormones and Cognition

There is a substantial body of literature that demonstrates the influence of sex hormones on cognitive processes. The clearest evidence of direct neurological effects of estrogen and progesterone was the discovery of their receptors in multiple sites of the brain (aside from the hypothalamus). Both estrogen receptors (ER-α and ER-β) and progesterone receptors (PR-A and PR-B) have been found in cognitively-relevant brain regions, such as the amygdala, hippocampus, and the prefrontal cortex [9,10]. Both hormones are lipophilic, which means peripheral levels can pass through the blood–brain barrier [11]. Furthermore, they influence synaptic formation through various signaling pathways and play critical roles in neuroprotection [9,10,12]. Rodent studies demonstrate ovariectomized rats had decreased dendritic spine density of neurons in the hippocampus and prefrontal cortex [13], with increased spinal density in these areas after exogenous estradiol administration [14,15]. Such studies also demonstrated an estrogen-enhanced correlation with performance on memory tasks [13,15]. Progesterone has been studied less extensively. While progesterone is considered neuroprotective for cognition after traumatic brain injury [10], rodent studies indicate it initially enhances, but then antagonizes estradiol enhancement of spinal density [16]. Estrogen and progesterone also interact with cognitively-relevant neurotransmitter systems, which include serotonergic, GABAergic, dopaminergic, and glutamatergic pathways—all of which play roles in executive function, learning, or memory [12].

Sex hormones have also demonstrated cognitive effects in human clinical studies. Estrogen depletion in natural or surgical menopause is potentially associated with cognitive impairment [17], and is a possible explanation as to why more women than men suffer, with greater severity, from neurocognitive disorders with postmenopausal onset such as Alzheimer’s disease [18]. There is also a large body of research investigating the effects of exogenous administration of sex hormones on cognition. This includes hormone replacement therapy in peri-menopausal and menopausal women [19,20,21] and oral contraceptive pills [22], as well as the effects of induced hypogonadism and hormone add-back therapy in young, healthy women [23,24]. This review, however, focuses on the influence of endogenous hormones in relation to the menstrual cycle and premenstrual mood disorders.

## 4. The Menstrual Cycle and Cognition

The menstrual cycle has long been a convenient model to examine the influence of endogenous sex hormones on cognitive functioning within a short time frame. Both “hot” (emotion-related) and “cold” (emotion-independent) cognition have been explored across the menstrual cycle. Emotion-related cognitive processes which have been studied include fear conditioning [25,26], e.g., emotional facial recognition (see review [27]) and emotion processing (see reviews [11,28]). Evidence suggests there are associations between emotion-dependent cognitive processes and menstrual cycle phase, with the luteal phase associated with poorer performance on emotion-related cognition. The evidence in relation to “cold” or emotion-independent cognition is less consistent [11,28] and is the focus of the current review. Most research into neurocognitive performance has been conducted with a hypothesis that stems from research into cognitive sex differences. Historically, it is claimed that men perform better in visuospatial tasks, while women demonstrate an advantage in verbal skills [29]. This theory was applied to the menstrual cycle by assuming that performance in these "sex dimorphic” tasks differed, at least in part, due to levels of sex hormones [30]. The hypothesis was that women would perform better in the follicular phase (when sex hormone levels were low) on visuospatial tasks that historically have been described as male favoring tasks. In contrast, performance on verbal tasks that are suggested to favor females would be improved in the luteal phase when levels of sex hormones were higher. Of the visuospatial task, the 3D cube mental rotation task, first devised by Shepard and Metzler [31], and adapted into a pen-and-pencil task by Vandenberg and Kuse [32], has been associated with the most consistent findings and the largest effect size [33] in relation to the research examining sex differences in cognition.

Studies of the menstrual cycle involve comparison of performance between at least two time points of the menstrual cycle. Sundström-Poromma and Gingnell’s comprehensive review in 2014 [11] of menstrual cycle studies in cognition concluded that there was insufficient evidence to support the hypothesis that male-favoring cognitive tasks would be improved in the follicular phase. Their review only included methodologically sound studies which conducted hormonal confirmation of menstrual cycle phase to streamline study quality. Across the 12 studies included for mental rotation, four studies supported the hypothesis [34,35,36,37] while the others had no significant findings [38,39,40,41,42,43,44,45]. However, multiple studies had methodological issues: three studies [36,38,46] had small sample sizes of under ten participants, significantly limiting their power; one study did not counterbalance the participants to prevent practice effects on results [34]; and two studies had cross-sectional study designs testing the participants only in one menstrual cycle phase [35,42], which can highly bias the results as inter-individual variability in baseline hormone levels and baseline cognitive capacity cannot be accounted for. Four of the studies compared performance between low and high estradiol [38,42,43,46], whether between the early and late follicular phase or between arbitrary points of different levels. This does not account for possible effects of progesterone and general menstrual phase. Four of the studies used the earlier version of the mental rotation test devised by Shepard and Metzler, instead of the Vandenberg–Kuse mental rotation task [34,40,41,43], which produces more pronounced sex differences. Studies that did find phase differences in mental rotation and other visual spatial tasks (including visual reproduction and spatial math tasks) all showed enhanced performance in the follicular phase [34,35,36,47,48]. Moreover, the two functional magnetic resonance imaging (fMRI) studies [38,45] in mental rotation showed there were brain activation differences in mental rotation between the sexes and enhanced activation for women in high estrogen phases (pre-ovulatory and mid-luteal) despite no significant performance differences. Estradiol concentrations were also correlated with increased activation.

For verbal skills, the studies included in the 2014 review were similarly inconclusive. The two main cognitive domains identified in meta-analysis as favoring females are verbal fluency and verbal memory [49]. Two of the six studies examining verbal fluency showed improved performance in periods of high estradiol [37,50], such as the mid-luteal phase or the late follicular phase, while the remaining studies showed no effect of phase [41,42,44,46]. Other studies of verbal skills have varied in the tests used, ranging from paragraph recall [47], implicit and explicit verbal memory tests [37], the California Verbal Learning Test [44], and verbal working memory [51]. No significant pattern has emerged in results. Results were subject to similar methodological inconsistencies—small sample sizes and two studies were cross-sectional [35,42]. The early follicular and mid-luteal phase were generally tested as comparisons. However, one study, which failed to detect a phase effect compared the early follicular phase and the days straight after ovulation, both phases when hormone levels are low [46], and thus their study design was not able to measure cognition at differing hormone levels.

The 2014 review showed that much of the evidence still required greater methodological rigor, and placed into question whether the menstrual cycle could sufficiently capture cognitive changes based on this historical hypothesis. An fMRI study by Pletzer et al. [52] demonstrated that despite a lack of menstrual cycle phase effect in performance on spatial navigation and verbal fluency tasks, there were differences in brain activation patterns during the follicular and luteal phases. Specifically, there was enhanced hippocampal activity during the pre-ovulatory, higher estradiol phase and enhanced fronto-striatal activation during the high progesterone luteal phase; these changes were irrespective of task. This study shows that sex hormones likely influence cognitive processes, but challenges the concept of ‘male’ or ‘female-favoring’ cognitive tasks over the menstrual cycle. Furthermore, contemporary research regarding sex differences in cognitive performance is moving toward a bio-psycho-social model [53,54] which involves socio-cultural factors as well as levels of androgenic sex hormones (e.g., testosterone). Preliminary studies also show that aside from performance, spatial cognitive strategies in the luteal phase may be more “egocentric” than “allocentric” [55,56], which is when spatial tasks are conducted in relation to one’s own perspective instead of cardinal direction (e.g., those of a compass). Such findings suggest that changes in gross performance across a menstrual cycle might not sufficiently capture the potentially subtle influence of sex hormones on cognitive abilities and cognitive strategies.

Aside from traditionally sex dimorphic tasks, menstrual cycle studies have also considered executive functioning tasks. Executive function is an umbrella term, referring to higher order cognitive processes, such as the capacity to monitor, organize, think flexibly, and plan. These processes are partly attributed to dopamine modulation within prefrontal brain regions, and sex hormone interactions with dopaminergic pathways [57]. Studies have similarly been inconclusive, mainly due to the heterogeneity of different tests used measuring varying aspects of executive function. The Wisconsin Card Sorting Test, which assesses shifting of mental set (or flexible thinking) and problem solving, had mixed results in two separate studies by the same authors. One study [58] showed better performance in the early luteal phase, when progesterone levels are at their highest, relative to late luteal phase and ovulation phase. The other study did not observe a menstrual cycle phase effect on this task [50]. Inhibition and selective attention performance varied depending on the task. Performance was shown to be impaired in the late follicular phase in a stop-signal task [59], improved in levels of high estradiol for the Stroop task [60], and improved in the late follicular phase for inhibitory return [61]. Thimm’s fMRI study using the Go/No-Go task showed that reaction times to the right visual field were faster in the early follicular phase (lower levels of estradiol) compared to the late follicular phase, but imaging did not produce clear findings to mirror the behavioral results [62]. High levels of estradiol have also been suggested to be related to improved working memory performance in verbal and spatial contexts [51,63].

Overall, the research is inconsistent regarding the menstrual cycle influence on cognition. In many of these studies, levels of estrogen, as opposed to progesterone have been explored as the modulator of cognitive performance. This is limiting, as progesterone in animal studies has an influence on cognition, with perhaps even an inhibitory effect on estrogen. Fluctuations of the menstrual cycle may also be too transient to reveal substantial findings, or methodological issues may cloud robust findings. Nevertheless, as Sundström-Poromma and Gingnell concluded in 2014 [11] and similarly in 2018 [11,28], methodologically-sound studies do not appear to demonstrate significant effects of menstrual cycle phase on cognitive performance.

## 5. Premenstrual Mood Disorders and Cognition

The studies of the menstrual cycle reviewed in Section 4 assessed groups of healthy women. Comparatively, as biological females with premenstrual mood disorders have a potential vulnerability to sex hormone fluctuations, it remains to be concluded whether changes in their cognition may be more detectable or pronounced in these clinical disorders. Women with PMS and PMDD have no absolute differences in peripheral hormone levels [7]. As previous menstrual cycle studies can only compare cognitive performance between absolute high and low hormone levels, investigating women with premenstrual mood disorders also considers the effect of sensitivity to hormone kinetics. Clinically, women with PMS and PMDD also describe symptoms in the late luteal phase that could be related to altered “cold” cognitive functioning, such as difficulty concentrating and impaired work productivity [7]. Symptoms such as affective lability, and a sense of being overwhelmed or experiencing a lack of control, could also involve alterations in cognitive-affective processes. Thus, it is imperative to understand if an objective cognitive change accompanies these symptoms.

Studies measuring cognitive performance in these clinical groups have similar study designs to previous menstrual cycle studies—they are repeated measure and compare cognitive performance between the follicular and the luteal phases. For the current narrative review, we considered observational studies evaluating cognitive performance in women with PMS or PMDD published in English after 1960. Studies were included if they involved: (a) a study group of premenopausal females experiencing symptoms of PMS or PMDD; (b) at least one clinical measure of cognitive performance assessing one or more of the following domains: attention, memory, working memory, executive function (including inhibitory control), verbal, or visuospatial abilities; and (c) the inclusion of a separate control group of naturally-cycling women without symptoms of PMS or PMDD or the evaluation of groups based on severity of PMS / PMDD. Exclusion criteria were (a) studies involving administration of additional medications or study conditions with potential for cognitive impact, unless meaningful analysis of baseline evaluations was reported, and (b) if full text of the studies were unable to be accessed. The most recent search was conducted on 23rd March 2020. Databases included MEDLINE, Scopus, and PubMED using the following key words “premenstrual syndrome”, “premenstrual mood disorder*”, “PMDD” or “Premenstrual Dysphoric Disorder” AND “cognition”, “cognitive performance”, “cognitive function”, “verbal”, “visuospatial”, “attention”, or “executive function”. Due to the small number of studies in the area, studies were not excluded on basis of methodological quality. While menstrual cycle studies in healthy women have primarily focused on cognitive tasks considered sexually dimorphic, studies on PMS and PMDD have focused more on cognitive domains such as executive functioning and attention, which are commonly affected in psychiatric disorders and significantly impact psychosocial functioning [64]. Cognitive performance between the subject groups and between phases was reviewed. 

### 5.1. Studies of PMS and Cognition

Five studies have examined cognitive performance in women with PMS, as outlined in Table 1. PMS is characterized in different ways across studies, so participant groups with PMS are likely to be broad and heterogeneous. Three studies demonstrated significant group differences, but reported that differences were small [65,66,67]. Of these studies, one study [66] included a healthy control group of naturally-cycling women who were not experiencing any PMS symptoms and the remaining two studies compared women with PMS by severity of symptoms instead of having an asymptomatic control group (e.g., “severe” vs. “mild”) [65,67]. Their findings suggest that increased severity of premenstrual symptoms could be associated with poorer cognitive performance. This implies that differences could be more pronounced in a PMDD group, who experience more clinically-significant symptoms.

Within the domain of attention, findings have been inconsistent. Attention span and working memory were assessed using the Digit Span task in three studies. This task asks participants to recall a string of numbers, then repeat the numbers in the same or reverse, backward order [68]. A computerized visual version of this task has been associated with slight (but statistically significant) differences between “high-symptom” and “low symptom” PMS participants in the luteal phase [65], with no significant differences across phases or between PMS and non-PMS groups in the two other studies [66,69]. The Trail Making Test, A and B assesses visual attention, psychomotor speed, and the capacity to divide attention (Trails B only). The task asks subjects to connect dots between numbers and letters—Trails A is numbers only, and Trails B alternates between numbers and corresponding letters [66]. Trails B, which is more complex and involves executive functioning skills such as ‘switching’ between numbers and letters, was one of few tasks to demonstrate group differences in Keenan et al.’s study [66]. This finding suggests that higher order functioning may be more impaired than other cognitive domains in women with PMS. However, performance in both the PMS and non-PMS groups remained in the 75th–90th percentile for their age, and a colored variation of the Trail Making task in Morgan et al.’s study showed no group differences [69]. Rapkin et al. used a non-specific psychomotor speed test which measured the speed subjects could find specified digits in lines of numbers, which also demonstrated no differences [70].

In relation to verbal memory, Keenan et al. found that women with PMS had impaired word recall and learning of new words below normal ranges in the California Verbal Learning Test (CVLT) that was non-phase dependent [66], but the other two studies using different tests (verbal paragraph recall [69] and the Hopkins Verbal Learning Test [67]), with larger samples showed no significant group differences. Slyepchenko et al.’s study [67] is the only study from the last decade, and found that PMS participants had poorer accuracy in the N-back task, a working memory task that is also impaired in PMDD studies reviewed below. Their study was novel by comparing groups only in the follicular phase. Their findings suggest that cognition may be affected outside of the symptomatic period, or they could be incidental given that their study cannot control for inter-individual variability in baseline performance.

### 5.2. Studies in PMDD and Cognition

Studies examining PMDD and cognitive performance are also limited, as summarized in Table 2. As a psychiatric disorder with a severe functional impact on interpersonal relationships, work and leisure, studies have examined if cognitive impairment exists in the luteal phase in women with this disorder. Studies from the 1990s tended to use the rationale that PMDD was a similar entity to major depressive disorder, and thus because cognitive deficits have been found in depression, similar deficits may be found in PMDD. At this time, PMDD was only part of the DSM-IV as a disorder requiring further research, and the first rank symptom was “markedly depressed mood” instead of affective lability, the first rank symptom in the DSM-5. More recent studies have focused on including cognition as part of a more holistic assessment of the disorder, or to identify cognitive deficits which may explain certain symptoms. For example, Yen et al.’s two studies in 2012 and 2014 [71,72] attempted to make links between working memory, irritability, and emotional regulation.

Within the domain of executive functioning and working memory, the most common task used, with the most consistent results, was the N-back task, assessing working memory [71,72,77]. This task involves asking participants to remember objects or letters they had seen a certain number of trials ago. Women with PMDD had poorer performance on the task compared to the control group in the luteal phase in all three studies, especially as the working memory demand increased. This suggests that working memory may be impaired in women with PMDD, and that the difference in performances in the luteal phase relative to the follicular phase becomes significant with increasing difficulty of the test. N-back performance also negatively correlated with irritability symptoms and positively correlated with functional impairment ratings in Yen et al.’s 2012 study [71]. This suggests that cognition may have a direct influence on psychosocial functioning. Similarly, Baller et al. showed that atypical activation of the dorsolateral prefrontal cortex in the N-back task in PMDD participants was positively associated with PMDD severity, earlier age of onset, and global functioning scores [77]. Furthermore, Yen et al.’s 2014 study found an association between a specific genotype of a serotonin receptor gene, HTR1A, and impaired performance in the N2 task [72]. Although a causal relationship cannot be established, this preliminary finding affirms a role for serotonin in PMDD and its interactions with cognition. 

However, other tests of executive function have had varying results. Impaired inhibition is a cognitive ability which could relate to impulsivity in PMDD [76]. When tested via a Go/No-Go task, women with PMDD showed significantly poorer performance in their luteal phase relative to the follicular phase compared to controls [72]. In contrast, Bannbers et al. found no group differences in fMRI despite brain activation differences [76]. The Stroop task also lacked group or phase differences in Resnick et al.’s study [73].

In the domains of attention and psychomotor speed, the Digit Span test and the Digit Symbol Substitution Test have been used. The Digital Symbol Substitution Test examines processing speed and attention [68]. Performance was decreased in the luteal phase for PMDD participants in Reed et al.’s study [75], even after averaging results across four sessions in the late luteal phase and four sessions in the follicular phase. Resnick et al. [73] found an interphase change in the PMDD participants, but results remained within normal range for women of that age. Their results were also similar for Trail Making Test A and B, with only small differences found. Although there was no control group, these results may suggest that changes are subtle, and not clinically significant.

Visuospatial tasks have rarely been assessed, with only Man et al. testing spatial working memory [74]. No differences were found between PMDD and control groups, but this was subject to a small sample size and both groups did make more errors in the luteal phase. For verbal skills, Reed et al. found that word recall in women with PMDD in the luteal phase was significantly worse [75], but Resnick et al. showed no group differences using the California Verbal Learning Test [73], even though it has shown to be impaired in PMS [66]. Tasks such as mental rotation and verbal fluency, which have been the focus of menstrual cycle studies, have not been tested before in PMS or PMDD. It is possible that using these tests, which may be sensitive to sex hormones, in these disorders may identify impairments not otherwise detected by other tasks.

### 5.3. Limitations of Clinical Studies and Scope for Future Research

PMS and PMDD studies have yet to be as methodologically rigorous as menstrual cycle studies in healthy women. Sample size is a clear limitation as the majority of the studies had less than 30 participants in each group. This limits the ability to extrapolate the findings to PMS or PMDD populations. The studies, by design, are also limited to testing participants over one menstrual cycle only. Both quantitative [78] and qualitative [79] literature describe that it is a fairly normal experience for women’s premenstrual symptoms to change one cycle to the next, and thus the study designs do not account for inter-cycle variability. Extending the studies however must be balanced with practice effects in repeat cognitive testing.

Furthermore, most did not use hormonal confirmation to confirm menstrual cycle phase except for Morgan et al. [69] who used a urine LH ovulation kit and two studies [72,75] taking serum hormone levels at each session. Instead, only retrospective assessments of participants’ previous cycle lengths were used to estimate when to test. This can be unreliable, and lead to assessments at inaccurate points of the cycle. Many women also do not spontaneously ovulate every cycle, which leads to symptom remission [80]. Serum hormone assays are standard practice for menstrual cycle studies in healthy women [11], and they are the most direct and reliable way to assess menstrual cycle phase and the hormone profile of individuals and subject groups [81]. Future studies should aim to include serum hormone assessment. Timing of testing in the luteal phase also varied slightly between studies. The main difference between studies was Yen et al.’s two studies testing participants “1 week before menses” [71,72], which is day 21 in a standard 28-day cycle, while the other studies generally tested between days 22 and 27 [65,66,69,73,74,75,76,77,82,83]. Day 21 is the peak of progesterone levels in the menstrual cycle, whereas testing a few days later is after its withdrawal, both of which could affect symptoms and cognitive performance differently. Man et al. did not indicate when testing was done in either phase [74]. Hormone kinetics are central to these disorders, thus timing of testing is pivotal for interpreting the mechanisms underlying cognitive changes.

Another limitation of PMS and PMDD studies is the varied use of symptom reporting and diagnostic tools. For a confirmed diagnosis, both PMS and PMDD require at least two cycles of prospective daily symptom reporting [84]. It is current consensus that the Daily Rating of Severity of Problems (DRSP) questionnaire [85] is the most widely used and validated for PMDD diagnosis [84]. None of the cognitive studies have used this questionnaire. Two studies [67,71] used a retrospective screening tool (Premenstrual Symptom Screening Tool) which has a lower specificity than the DRSP [86], and Diener et al. used an outdated retrospective Moos Menstrual Distress Questionnaire devised in 1968. Therefore, these studies are subject to recall bias and inaccurately defined subject groups. The other studies did use prospective ratings, but the lack of consistency of standardized questionnaires used (e.g., Cyclical Diagnoser scale, “daily rating diary”) means varying subject groups may have been captured. This varied stringency for classifying clinical groups was therefore present for control groups too. In Slyephchenko et al.’s [67] and Man et al.’s [74] study, control subjects were acceptable as long as they did not meet PMDD criteria, whereas Yen et al. [71,72] and Reed et al. [75] had specific criteria for controls (e.g., having fewer than *x* criteria met for PMDD diagnosis). Furthermore, combining both mild and moderate symptomatic women heterogeneously into one clinical group in Resnick et al.’s study [73] may have masked more significant group differences. As up to 80% of women experience at least psychological or physical symptom before menses [87], normal physiological changes must be differentiated with clinical premenstrual disorders in order to compare the cognitive profiles of two distinct groups.

In light of these methodological concerns, their findings are therefore only preliminary. Future research should thus focus on developing robust study designs, as well using a broader range of cognitive tasks. Investigating cognitive aspects of PMS and PMDD enables understanding of its symptomology as well as clinical applications of diagnosis and management. This could also lead to therapeutic studies exploring hormone therapies and cognitive outcomes in women with PMS and PMDD.

## 6. Conclusions 

The menstrual cycle is a pivotal model to study how sex hormone fluctuations can alter cognition. Studies examining the menstrual cycle in healthy women have been unable to show consistent associations between cognition and menstrual cycle phase. It may be that the sex difference hypothesis behind many of these studies may be outdated, or any changes that do occur are too transient over a monthly cycle to be detectable in gross performance changes (instead, cognitive strategies may differ across menstrual cycle phase). Regarding premenstrual disorders such as PMS and PMDD, although methodologically-sound studies are limited, current evidence suggests that there may be cognitive deficits in some aspects of executive functioning in those who are most symptomatic (women with PMDD). This suggests that while sex hormones are influencing cognitive processes over the menstrual cycle in both healthy and premenstrual disorder groups, the effects may not be clinically relevant until severely symptomatic. Nevertheless, it is important to consider the observational nature of these studies means no causation of any cognitive change observed can be confirmed—it is unclear whether the negative experience of mood and psychological symptoms affects cognitive abilities, or whether it is an underlying hormonal dysregulation directly impacting cognition. Research into PMS and PMDD remains in its infancy, and thus further study into these clinical disorders should be the focus. Further study would ideally incorporate the methodological aspects of previous menstrual cycle studies, as well as examine cognitive tasks that may be sensitive to sex hormones.

## Figures and Tables

**Table 1:** Cognitive studies in premenstrual syndrome (PMS).

Author	Year	Sample Size	Cognitive Task	Hormone Assays	Group or Phase Differences
Rapkin et al. [70]	1989	10 PMS 9 controls	Mental arithmetic, perceptual speed test	No	No significant group or phase differences
Diener et al. [65]	1992	8 ‘high symptom’ PMS, 8 ‘low symptom’ PMS	Digit Span, letter detection	No	↓ performance in “high symptom” group in luteal phase
Keenan et al. [66]	1992	14 PMS 10 controls	California Verbal Learning Test, Stroop task, Trails A and B, verbal fluency, finger tapping, grip strength, digit span	No	Significant group, non-phase-dependent impairment in California Verbal Learning Test, ↓ performance in Trails B task in the luteal phase (performance still within standardized range for healthy females)
Morgan et al. [69]	1996	30 PMS 31 controls	Color trail-making, figure maze test, block design, figure memory, verbal paragraph recall, visual memory, digit symbol test, digit span	Urine LH ovulation test	No significant group or phase differences.
Slyepchenko et al. [67]	2017	13 Mod/Severe PMS 27 Mild/no PMS	Hopkins verbal learning test, letter–number sequencing, N-back task, finger tapping, emotional Stroop task	No	↓ performance in N-back task in the follicular phase of moderate/severe PMS group.

“↓” means “Reduced”.

**Table 2:** Cognitive studies in premenstrual dysphoric disorder (PMDD).

Author	Year	Sample Size	Tests Used	Hormone Assays	Results in PMDD Participants
Resnick et al. [73]	1998	19 PMDD 18 mild–moderate symptoms	Digit Symbol Substitution Test, Grooved pegboard test, Digit vigilance test, Trail Making Test,, Stroop task, California Verbal Learning Test	No	↓ psychomotor index score in the luteal phase relative to follicular phase
Man et al. [74]	1999	10 PMDD 10 controls	Tower of London, spatial working memory	No	No significant group or phase differences
Reed et al. [75]	2008	14 PMDD 15 controls	Word recall, digit recall, Digit Symbol Substitution Test, divided attention task	Each session (8 times)	↓ performance in all tasks except divided attention in the luteal phase
Yen et al.[71]	2012	60 PMDD 60 controls	N-back task	No	↓ performance in the luteal phase for all N-back tasks, ↓ performance in the luteal phase relative to follicular phase (N3 task only)
Yen et al. [72]	2014	59 PMDD 74 controls	N-back task Go/No-Go	Once each phase (2 times)	↓ performance in the luteal phase relative to follicular phase in both tasks
**Imaging studies**					
Bannbers et al. [76]	2012	14 PMDD 13 controls	Go/No-Go	No	No significant group differences
Baller et al. [77]	2013	PET: 15 PMDD, 15 controls; MRI: 14 PMDD, 14 controls	N-back task	No	Overall ↓ performance compared to controls (phase not studied as artificial hormone conditions used)

“↓” means “Reduced”.
